# Periscope

**Published:** 1890-03

**Authors:** 


### PERISCOPE.

### MEDICINE.

**Tetany after Extirpation of the Thyroid.**—Eiselberg, at the recent Congress held at Heidelberg, brought forward some statistics to show the frequency with which tetany occurred after the extirpations of the thyroid carried out by Billroth. The operation was performed in fifty-two cases, and of these twelve were affected by this disorder, usually after some prodromal symptoms. Two were but slight, while six were moderately severe. In a woman, nine years after extirpation, frequent attacks occurred. She always suffered from bronchial catarrh, with tough expectoration; but the mental condition was normal. Four of the cases were fatal—one after four weeks, a second after seven months. In every case the thyroid was excised for a goitre, which caused difficulty of respiration, two of them being cancerous. All were in females between the ages of twelve and sixty-four; but this is to be considered merely a coincidence. Morphia and chloral relieved the slight cases, severe ones not being at all influenced. As a prophylactic, total extirpation of the gland should be avoided where possible. From experiments in cats, simple complete excision is always followed by tetany and death in seven to forty days; unilateral extirpation (in sixteen instances) did not show tetany as a result. On eight occasions the gland was removed in two portions at different times; one half was cut out and transplanted into a fold of the omentum. After three days to three weeks, the other half was excised, when all the animals died, with the exception of one, in which the transplanted piece of gland had become organised in the omentum. Eiselberg believes the function of the gland to be, that it renders innocuous a mucinoid substance in the body which occasions a form of poisoning. Thus, Wagner, by injecting mucin from the salivary glands of oxen into healthy cats, produced tetany; and the reason why so many people escape this affection after extirpation of the thyroid is, that there are usually accessory glands having a similar function.—*Berliner klinische Wochenschrift*, No. 43, 1889. D. R. P.

**Angina from Affection of Vagus.**—Professor Obolensky relates a case which may be looked upon as an illustration of the above. An officer, aged forty-two, complained of pain in the sternal region, palpitation and breathlessness. In severe attacks the pain extended into the upper extremities and intercostal region, and was induced often on exertion even such as kneeling. Sometimes an attack seized him in sleep, and he was obliged to spring out of bed, with pain and dyspnœa. At other times the drinking of tea, or the mere process of eating, led to one. They usually lasted five to ten minutes; in some cases half an hour. They had begun first of all four months before and one month after a severe shaking, received in a railway-collision, from which, however, he had fully recovered. An examination of the chest yielded nothing. In an attack which came on as soon as he moved a few steps, the respiration rose from 24 to 36, and the pulse from 72 to 100. The cervical portion of the left pneumogastric was tender on pressure. The previous history showed that at nineteen the patient suffered from a paralytic affection, which was cured by iodide of potassium, and three years after he was again threatened by the same trouble, which yielded to similar treatment. He suffered from pains in the lower extremities; and his father had formerly been the subject of ulceration in various parts, more especially of the mouth. All these facts supported the view that one had to deal with a syphilitic process. Iodide of potassium was accordingly given, and within a month the patient was discharged well.—*Berliner klinische Wochenschrift*, No. 52, 1889. D. R. P.

**Epistaxis Premonitory of Interstitial Nephritis.**—Gaucher insists on the necessity of examining the urine of all patients liable to frequent attacks of epistaxis, as we shall find in some of the cases that we have to do with kidney-mischief.—*Revue de Laryngologie*, May 1st, 1889. B. J. B.

The next four abstracts are translated from the *Deutsche Medizinal-Zeitung* and *Allgemeine medicinische Central-Zeitung:*

**The Infectious Nature of Fibrinous Pneumonia.**—G. Lipari (*Centralblatt f. klinische Medicin*, No. 50, 1889) confirms our previous knowledge of Fränkel's pneumococcus, and shows experimentally the influence of cold as a cause of fibrinous pneumonia. The endo-tracheal injection of pneumonic sputum, or of pleural exudation from an animal dead of pneumonia, gave a negative result; for out of eleven guinea-pigs and rabbits only two died, one only having foci of consolidation. Quite different results followed when the animals, either before or after the injection, were made warm by exercise, and then placed for ten or twenty minutes in a bath of 3° Cent., or had ether applied to the shaven chest. Out of five guinea-pigs and three rabbits treated thus, three of the former and all the latter died with true pneumonic infiltration. The author considers that cold causes a paralysis of the ciliated epithelium of the bronchi, and by congestion a swelling of the mucous membrane, and that through these two factors the infective material sinks into the alveoli. This explains the effect of cold in the production of pneumonia in man, though numerous pneumococci are present in the human mouth during health. G. P.

**Actinomycosis.**—According to Ernest Partsch (*Volkmann's Sammlung*, 306-7), the human body shows the purest form of actinomycosis infection, and is affected only through the production of a granulation tissue which is marked by softness, a tendency to bleeding and to fatty degeneration. The accompanying suppuration is brought about, not by the fungus, but by the introduction of the coccus pyogenes at the same time. The parasite spreads from the original focus, forming indurations which develop into tumours, and is stopped neither by fasciæ nor bones. It never propagates itself by the lymph-channels, but by destruction of the vessel-walls easily reaches the inner organs through the blood-stream.

The swelling of the lymph-glands is of a purely inflammatory nature. Actinomycosis, as such, causes no lymph-gland metastases. It differs in this from syphilis and tuberculosis. It does not tend to a dyscrasis. It is at first a purely local affection, and its extension depends on the entrance of the poison into the blood-channels. It is not hereditary, and is to be placed among chronic infective diseases. Its product is an infective granulation tumour. As in animals, infection occurs through the food and inspired air. Communication from beasts to men is not yet proved. The fungus lives as a parasite on grain, and fastens on the teeth or tonsils or enters the lungs. From the mucous membranes it enters the body cavities, and occasionally it may enter through a wound. As to the treatment, favourable results have been obtained where thorough surgical extirpation has been possible. G. P.

**On the Influence of Human Gastric-juice on Pathogenic Germs.**—Kurlow and Wagner (*St. Petersburger medicinische Wochenschrift*, No. 42, 1889) describe certain experiments made by themselves, and come to the following conclusions:

(1) Certain microbes which are wont to live in the stomachs of healthy persons are not affected, but those which enter with the food and saliva are not in a position to continue their existence in normally acid stomachs.

(2) The gastric juice is a very powerful destroyer of pathogenic germs.

(3) When it acts normally its destructive power is only escaped by microbes with strongly resisting spores, such as the tubercle and anthrax bacilli, and sometimes staphylococci. Those which have not such spores die in less than half an hour in normal gastric juice. Such are those of typhoid, cholera, tetanus, and B. pyocyaneus. A similar destructive power is found in the intestines, as is shown by certain failures in inoculation with the cholera bacillus; but on this further researches are being made. G. P.

**The Influence of Ventilation on Organisms Suspended in the Air.**—Ric. Stein, in the Hygienic Institute at Breslau, has made certain investigations on this point, which have led to remarkable results. In quiet air in a chamber the particles containing bacteria fall to the ground. The air will in one or two hours be completely free of germs. If the floor be then washed with an antiseptic solution, and the furniture and similar articles wiped with a cloth wrung out in it, the chamber can be considered as disinfected.

Ventilation, which consists in the renewal four times an hour of the air contained in a chamber, has no special influence on the removal of germs floating in the air. Only in a strong draught is a quick and complete removal of germs from the air to be obtained.

The use of a spray appeared to have no effect in purifying the air. Even under strong draught, neither the floor, carpets, nor furniture were purified. The practice of hanging up infected clothes and bedding in the air is useless, unless they are at the same time beaten and brushed, an operation dangerous to the workmen and neighbourhood.—*Allgemeine medicinische Central-Zeitung*, No. 4, 1890. G. P.

### THERAPEUTICS.

**Recent Treatment of Pulmonary Tuberculosis.**—Dr. Paul Chiron gives a *resumé* of the various new medications advocated for pulmonary phthisis, and the results obtained:

**I. Medicated Inhalations.**—Inhalations of carbonic acid, according to Nothnagel and Nosbach, do harm; those of benzoate of soda are useless. The results obtained by inhalations of iodoform and of terebene are contradictory. Sulphurous acid has been tried by Dujardin-Beaumetz, with benefit as regards cough and expectoration.

Fluorhydric acid inhalations have been more extensively employed. M. Raimondi treated 128 patients, of whom twenty-eight were cured (disappearance of bacilli), eighteen improved, and five died.

Gœger obtained, on the whole, favourable results in seventeen cases treated. He found, however, that in twelve patients who gained weight, this gain bore no constant relation to amelioration of the local changes. When there is tubercular laryngitis, feeble solutions must be used, and the inhalations be few and far between on account of the risk of exciting inflammatory changes.

Andollent considered that the inhalations had more effect on the general state than on the local lung-lesions.

Dr. Burnet has not obtained very encouraging results from inhalations of fluorhydric acid; beyond improved appetite, he noted no positive benefit from the treatment, and in some patients it appeared to provoke diarrhœa.

Dr. Tisnè found there was improvement at first; but this was not maintained.

Gilbert has studied the effects on thirty patients, and thinks that fluorhydric acid is the best of the new medications. He found, however, that the bacilli never disappeared from the sputum, and that the physical signs underwent little change. These inhalations are contra-indicated in asthma and even in emphysema, and should be suspended if hæmoptysis occurs.

These different observations show that the number of cures is not greater from the employment of fluorhydric acid than it is from ordinary methods of treatment. The one constant result is, the rapid improvement of appetite: the importance of this is not to be undervalued in phthisis; but further experience has not confirmed the early enthusiastic reports as to the number of cures effected.

The method of Bergeron, of gaseous rectal injections, seems in certain cases to improve the general well-being and appetite; but, as this can be obtained by less troublesome and disagreeable methods of treatment, it has fallen into disuse.

**Subcutaneous Injections.**—Gilbert injected eucalyptol subcutaneously, with no advantage to the patients: the injections gave rise to considerable pain, and in one patient a small fluctuating swelling remained at the site of injection; and, on incising this six weeks afterwards, he drew out a fluid similar to that injected, and of about the same quantity.

Dr. Shingleton Smith has employed injections of camphorated phenol, with some success.

**Intrapulmonary Injections** have been almost given up, even by the promoters of this form of treatment: no advantage, not to be obtained by other less dangerous methods of treatment, has been gained from their employment. Meanwhile, M. Fernet has in four patients injected a solution of camphorated naphthol $\beta$, with improvement in the physical signs in three of them.

**Internal Remedies.**—Dochmann and Mastell have used calomel, with, as a constant result, diminution of fever, night-sweats, cough, and cessation of diarrhœa.

Sticker employs iodide of potassium in cases of fibroid phthisis, and those in whom there is emphysema. Where the bronchial mucous membrane is especially affected, iodide should not be given, but creasote and the balsams employed. Contra-indications to the use of iodide of potassium he finds in hæmoptysis, laryngeal lesions, and œdema of glottis; interstitial nephritis or amyloid disease of kidney; iodism, purpura, pemphigus, and cardiac weakness.

Laskoff claims that he has cured ninety out of 112 tuberculous patients by administering an infusion of homeriana, a polygonaceous plant growing wild in Russia.

As to super-alimentation, in spite of the undoubted benefit derived from it in certain cases, it has not come into general use. Most patients find it difficult to continue to swallow for any length of time the enormous doses of meat-powder required: some of these powders are the reverse of palatable either in taste or odour. By choosing, however, the best kinds and substituting other foods for them when the patient has recovered a little appetite, good results may often be obtained.

Since the publication of Weigert's observations, inhalations of hot air have been largely used. The patient inspires air heated at first to 100° C.; after two or three days, to 150° C. The hot air is inhaled at first for half an hour twice daily, increasing the period to two hours twice a day, or even to six hours daily. The patient must breathe deeply; and should remain quiet for half an hour after each *séance*. The therapeutic effects are: increased strength; diminution of dulness and of râles; diminution in number, or even disappearance, of bacilli. Expectoration is increased at first; afterwards diminishes. The treatment is contra-indicated when there is hæmoptysis, or very profuse expectoration, with fever and loss of weight. The inhalations are useless in advanced phthisis complicated with syphilis, albuminuria, or intestinal ulcerations.

Finally, as to symptomatic treatment, iodoform is useful in hæmoptysis; antifebrin is perhaps the best antipyretic; and sulphonal has been employed with success in night-sweats.—*L'Union médicale*, Aug. 27th, 29th; Sep. 3rd, 1889.

J. M. C.

**Creasote in Pulmonary Phthisis.**—It is found that the presence of creasote in culture-fluids, in the proportion of .8 in 1,000 parts prevents, and in the proportion of .06 in 1,000 retards, the development of the tubercle-bacillus. Bouchard has determined by experiment that fifteen grammes of creasote in the day is a lethal dose for a man of sixty kilogrammes weight; while in practice a daily dose of more than three grammes—forty-five grains—is never necessary. According to him, the *rationale* of the use of creasote in phthisis lies in the following figures: .06 in 1,000 of creasote in nutritive media prevents the development of the bacillus; while to kill an animal by intravenous injection, it must be present in the proportion of .17 in 1,000 of the body-weight. Theoretically, then, it ought to be possible to attack the bacillus by a quantity of creasote which, while it restrains the activity and development of the microbe, is only the third part of the dose poisonous to the patient. M. Bouchard gives a daily dose of twelve to fifteen grains. For small doses he gives a pill which contains 1½ grains creasote, every two hours, to the number of eight or ten in the day; for larger doses he administers the creasote in solution in cod-liver oil, each tablespoonful containing a little more than 11½ grains. One or two spoonfuls are taken night and morning. It is necessary to be very careful not to cause irritation of the stomach.

Subcutaneous injections of the oily solution, which are a little painful, are employed by M. Gimbert; he and M.

Tapret also employ methods, by which the atmosphere in which the patient lives is charged with creasote. The results obtained seem not to be better than when it is given by the mouth. Bouchard claims that within eight to fifteen days expectoration, cough, and fever diminish, appetite and strength improve, and night-sweats cease. The patients gain in weight and the physical signs improve. In thirty-five per cent. of patients there was improvement; in twenty per cent. of cases in the first and second stages the symptoms, both local and general, disappeared. Unfortunately several of the cases afterwards relapsed.

Many other authors report encouraging results. Relying on his own and Lesguillon's observations, Mignon (*thèse de Paris*) states that creasote has a local antiseptic rather than an anti-bacillary action, and thus hinders the fermentative processes which so often give rise to fever in the phthisical.

Stachiewicz has employed intra-pulmonary injections: these injections are useful when they destroy the diseased tissues, and thus limit the morbid process. His conclusions are, that if only one lung is affected and there are cavities, the injection of creasote causes a rapid destruction of the diseased tissue; if the patient is inclined to hæmoptysis, or if there is extensive tubercular infiltration, the injections are contra-indicated. After each injection, while cough and expectoration are increased, the temperature remains unchanged.

Van der Veset recommends the following preparations, as more palatable than creasote in oil:

I. Oil of sweet almonds, creasote, part. æq.; five to ten drops in two tablespoonsful of milk, three or four times a day.

II. Creasote, six grammes; tincture of nux vomica, eight grammes. Eight to ten drops in two tablespoonfuls of sweetened water, before meals, three to five times a day.

Struecker thinks that creasote is especially indicated in cases of "caseous puenmonia," but that it is contra-indicated in cases of advanced phthisis, or when there is tubercular ulceration of the bowels, or lardaceous degeneration.

Sommerbrodt gives the results of the administration of creasote in nearly 5,000 cases of phthisis. He never exceeded a daily dose of about eleven grains. Most of the patients improved; in recent cases, with lesions of limited extent, the results were surprisingly good; in advanced cases there was no improvement. Tubercular laryngitis was cured in several instances.

Fraentzel does not give creasote to febrile patients; when bacilli were numerous in the sputum, no diminution of their number was observed under treatment. Nausea, vomiting, loss of appetite, gastralgia, fits of coughing, diarrhœa, and invincible repugnance to the drug were sometimes noted, and compelled him to discontinue it.

Sublinski confirms Fraentzel's results; he observed no modification in the number of bacilli, and does not regard hæmoptysis as a contra-indication to the use of creasote.

Sahli, and later Fraentzel, have used guaiacol, the active principle of creasote.

From these researches, creasote appears to be of advantage in many cases of phthisis, especially in recent ones; in very acute cases it is useless. It probably has an anti-bacillary action, and further certainly hinders the process of putrid intoxication. It must be given in sufficient doses, twelve to fifteen grains daily, and continued for a long time: the least irritating preparations should be chosen.—P. RODIAS, *L'Union médicale*, May 28th, 1889. J. M. C.

**Exalgine.**—Exalgine is chemically ortho-methylacet-anilin, *i.e.* a methyl derivative of acetanilin or antifebrin. Exalgine, like the other bodies of its class, has antiseptic. antipyretic, and analgesic properties; the latter predominate. It relieves all kinds of neuralgic pains when given in one single dose of $3\frac{3}{4}$—6 grains; or 6—11 grains may be given in the 24 hours, in two separate doses. Its advantages over antipyrin are, that it is more effectual, and a smaller dose is efficient, while it produces no untoward results; slight erythema in one case is the only instance of toxic action hitherto noted. Like antipyrin, it diminishes the amount of the urine and of the sugar passed in diabetes.—DUJARDIN-BEAUMETZ, *Gazette des hôpitaux*, March 26th, 1889.

J. M. C.

**Sulphurous Water in Laryngeal Tuberculosis.**—Dr. J. Chardrac finds that the usual treatment at any watering-place where there are natural hot sulphur springs is very bad for this condition, and warns us against Cauterets, Eaux-Bonnes, and such places, as patients always return with the larynx showing signs of increase of irritation, ulceration, and so on.—*Revue de Laryngologie*, April 15th, 1889.

[Patients with phthisis of lungs or larynx are nowadays so frequently sent out of England, and by preference to some part of Europe, that it is necessary to remember that a health-resort which may be excellent for the pulmonary condition may be quite the reverse for the inflamed, infiltrated, and ulcerated larynx. For instance, Davos and Engadine Kurorts are, in my opinion and from personal observation, most unsuitable for cases of phthisis laryngea, the dry, rarefied air being most irritating; in fact, " Davos throat," *i.e.* congestion of larynx and pharynx, is one of the inconveniences that many of the patients who reside there have to put up with, even though the throat was quite healthy before leaving home.] B. J. B.

**On the Treatment of Exophthalmic Goitre.**—Dr. Th. Schott, after alluding to the action of digitalis in controlling the palpitation of the heart; large doses of quinine, as recommended by Friedreich; iodides, as used by Trousseau, from which we undoubtedly derive help in these cases; belladonna, which has a quieting action on the nervous system—all of which are uncertain in this disease,—passes on to the consideration of some other less known methods of treatment:

(1) *Electricity.*—He recommends a weak constant current through the cervical sympathetic, or even the medulla, as giving us most satisfactory results.

[I have just now a most interesting case, in which there has been decided decrease in the volume and lessening of pulsation and hardening of the enlarged thyroid gland under frequent application of the constant current.]

(2) *Gymnastics.*—This was found to decrease the pulse, and to slow the breathing and make it more rhythmical.

(3) *Baths.*—Trousseau's recommendation of cold douches Schott thinks ought to be carried out with caution, as they are rather severe. He considers that those waters that contain carbonates and iron, *e.g.* Schwalbach, Pyrmont, &c., and also the hot baths of Nauheim, Rehme, &c., which are rich in carbonic acid, are very beneficial. Friedreich advises against sea-baths and residence at the seaside.

(4) *Elevated health-resorts.*—Schott recommends an elevation of 3,000 to 5,000 feet above sea-level, such as Engelberg, Black Forest, &c.—*Deutsche Medizinal-Zeitung*, April 18th, 1889. B. J. B.

**Palliative Treatment of Epithelioma of Larynx and Pharynx by Tincture of Thuja Accidentalis.**—Baratona has tried this drug in epithelioma affecting the larynx and pharynx, in doses of twenty drops to one drachm of the tincture, in the form of spray or otherwise applied to the parts. The action is beneficial in papilloma of the ear and nose, but it is useless in carcinoma and sarcoma. In two cases of laryngeal epithelioma the growths appear to have been arrested by the drug.—*La Pratique médicale*, No. 24, June 12th, 1889. B. J. B.

**Some New Remedies in Diseases of the Throat.**—Millican finds tincture of Aesculus hippocastanum, in three-drop doses in water every three hours, good for chronic laryngeal catarrh, with muddy colour and sluggish movement of the vocal cords. He also recommends two or three drops of tincture of arnica for throat-fatigue, with very little abnormal objective appearances. Bichromate of potash, in a dose of three drops of a solution of one grain in two ounces, was found of use in chronic laryngeal and pharyngeal catarrh, with thick, stringy mucus and irritative cough, and also when the capillaries of the pharynx and larynx are brightly injected, whether in acute or chronic conditions.—*Journal of Laryngology and Rhinology*, May, 1889. B. J. B.

**Treatment of Gall-stones.**—Rosenberg calls attention once more to the use of olive oil in this connection. His experiments show that after the inception of a large amount of oil or fat, a great increase in the secretion of bile, as well as a considerable dilution of it, takes place. As much as six ounces of olive oil may be given, and in many instances the addition of menthol, cognac, or finely-ground yolk of egg may be necessary to mask such a large quantity of fluid fat.—*Berliner klinische Wochenschrift*, Nos. 48 and 49, 1889. D. R. P.

**Salicylate of Soda.**—In the same article Rosenberg dwells on the value of this salt in cholelithiasis. In one case it was given in 15-grain doses, three times daily, in half a litre of hot water, and twice a day as a high rectal injection of 75 grains dissolved in a litre of warm water. The result was excellent—twenty-four hours after, gall-stones appearing in the stools. Stiller (*Wiener medizinische Presse*, No. 1, 1890) confirms this experience, using the remedy four times daily in 8-grain doses dissolved in soda water, sometimes combining with it ext. belladonnæ gr. $\frac{1}{6}$; he proposes to try it, perhaps in smaller doses in catarrhal jaundice. The same writer maintains that salicylate of soda possesses a strong diuretic action, and is very useful in promoting the rapid absorption of serous pleural exudations. Wertheimber (*Münchener medicinishe Wochenschrift*, No. 44, 1889) claims for it, from an experience of three cases, an almost specific effect in the treatment of universal pruritus. It was administered in doses of two tablespoonfuls of a three per cent. solution three times a day, and was followed, not only by relief, but by actual cure. D. R. P.

**Sweating Feet.**—For the treatment of this painful affection the following is recommended as a dusting powder (*Wiener klinische Wochenschrift*, No. 34, 1889): Acid. salicylic, 5; aluminis, 2; acid. tannici, 3; and amyl. trit., 90 parts. With regard to the use of chromic acid solution, which is used so generally for the same purpose in the German army, Prof. Kobert has pointed out the risk of the acid acting upon the small excoriations between the toes, and leading to serious ulceration.

A further method, recommended by Legoux, is as follows: By means of a brush or feather, the sole and parts between the toes are painted with the following mixture: Liq. ferri sesquichl., 3; glycerin., 1; and ol. bergamot., 2 parts. In a few days the sweat and odour disappear. D. R. P.

**Vomiting of Pregnancy.**—Gottschalk (*Berliner klinische Wochenschrift*, No. 40, 1889) was successful in a very obstinate case of vomiting, where even cocaine failed, by the administration of menthol in the following mixture:

Menthol ... ... ... ... ... ... ... grs. xv.

S. V. R. ... ... ... ... ... ... ... ʒ vi.

Aq. destill. ... ... ... ... ... ... ad. ℥ vi.

A tablespoonful every hour.

After the third dose the vomiting ceased; next day the frequency of administration was lessened; and a few days later the patient was discharged. The addition of some cognac helps the solution of the menthol. Weiss (*Therapeutische Monatshefte*, No. 1, 1890,) prefers the following formula:

Menthol ... ... ... ... ... ... ... grs. xv.

Solve in

S. V. R. ... ... ... ... ... ... ... ʒ vi.

Syr. sacchar. ... ... ... ... ... ... ℥ i.

A teaspoonful every hour. D. R. P.

**Orthin.**—It would be very desirable, in the interests of both patient and practitioner, were the procedure of Professors Kobert and Unverricht in the investigation of this substance generally adopted. The former, from theoretical considerations, suggested orthin—a hydrazin derivative—as an efficient antipyretic. The latter, in the same journal (*Deutsche medicinishe Wochenschrift*, No. 2, 1890), as a result of clinical trials, declares the drug of his colleague to be, of all the antipyretics yet introduced, the most dangerous and useless!
D. R. P.

**Theobromin.**—This is an excellent diruetic, strongly advocated by Gram of Copenhagen (*Therapeutische Monatshefte*, No. 1, 1890). As theobromin-sodium-salicylate, it is readily absorbed, and has no action on the heart. The diuresis is marked. The usual daily quantity given is 90 grains, in single doses of 15 grains. D. R. P.

The five following abstracts are translated from the *Deutsche Medizinal-Zeitung* and *Allgemeine medicinische Central-Zeitung* :

**On the Mechanical Treatment of Erysipelas.**—Dr. Wölfler has shown beyond question, by a new series of experiments, the efficacy of strips of plaster in the mechanical treatment of erysipelas. These can be fastened firmly, not only on the extremities, but also on the trunk and even on the shaven head. Care has to be taken that the strips, which must form a complete circle, do not become loosened, or are fastened again if they do. If the erysipelas has not yet reached to the strips, there is no special danger; but if that is the case the plaster is apt to soften and become loose in consequence of the increased temperature. If the erysipelas pass the strip with undiminished redness, it has certainly been badly fastened; but even across well-fastened strips, a much fainter pale-red colour may extend for a finger's breadth. For greater certainty, one can place a second strip at some distance from the first.—*Wiener klinische Wochenschrift*, Nos. 23, 24, and 25, 1889.

Kroell uses a guttapercha band in place of the plaster. Haberkorn gives internally benzoate of soda, four or five drachms daily; and Fox applies a compress moistened with a 1—4 per cent. solution of creasote. Calvelli paints the parts with one per cent. solution of picric acid, five or ten times a day.—*Deutsche Medizinal-Zeitung*, No. 85, 1889.

Dr. C. Rosenthal describes a combination of Pirogoff and Hueter's methods, which has succeeded when every other plan had failed. Pirogoff administers 2½ grains of camphor every hour or two, with hot drinks and other helps to perspiration. By Hueter's method the micrococcus is directly attacked by injections of three per cent. carbolic acid in the spot where it is, and a barrier, as it were, placed round the disease.

Favourable results are only obtained with cases taken at an early stage. Here numerous injections are made, with a disinfected Pravaz' syringe, in the subcutaneous tissue of the affected part of the skin. If it is about the size of the palm of the hand, six or eight injections are made at a centimetre apart. In strong people these may be increased up to twelve or fifteen, without any sign of carbolic acid poisoning. If the disease is widely spread, only the worst parts are to be thus attacked, and after twelve or twenty-four hours a new series of injections is to be used. In early cases, treated actively, Hueter entirely removed the disease within twenty-four or forty-eight hours.—*Berliner klinische Wochenschrift*, No. 42, 1889.

J. Koch employs an ointment, consisting of creolin one part, lanolin ten parts, and iodoform four parts. This is painted once a day on the affected spot and for a finger's breadth beyond it. Guttapercha tissue is placed over it. Very good results have been obtained by this plan.—*Wiener klinische Wochenschrift*, No. 27, 1889. G. P.

**On Disinfection of the Genital Canal.**—The results of Drs. Doderlein and Günther as to reliable disinfection are:

(1.) Washing out the vagina with sublimate or carbolic lotion is not a certain method of disinfection.

(2.) The introduction of the finger, anointed with vaseline, hinders the effect of a washing-out.

(3.) If the vagina be washed out with sublimate or carbolic lotion so thoroughly that it is free from germs, it becomes dry and chapped.

(4.) Creolin-mollin is recommended instead of vaseline. By wiping and washing out the canal with a two per cent. Creolin lotion, it can be made aseptic without interference with the integrity of the mucous membrane.

On the ground of these experiments, disinfection was exclusively carried out in the Leipsic Clinic by three per cent. creolin emulsion, and very favourable results obtained. The hands were washed with soap and a brush for five minutes in hot water; the soap was then washed off in fresh water, and the hands lastly disinfected with warm sublimate or carbolic lotion. The canal was disinfected before each examination and after the birth, the labia being pressed together to retain the fluid for some time, so that every crevice and fold was purified. This was completed by anointing the vagina with mollin.

It is only fair to mention that these results contradict the successful ones of Steffeck, in Giessen, who found creolin far inferior to carbolic and sublimate disinfection.—*Archiv für Gynäkologie*, xxxiv., I., 1889. G. P.

**Antipyrin.**—Torpid ulcers of the leg become healthy and quickly cicatrize under antipyrin dressing. The pain-destroying and antiseptic qualities of antipyrin have been shown by Neudörfer, and are corroborated by Schreiber, who records that some extremely painful and obstinate varicose ulcers in a man seventy-two years of age healed quickly when daily dusted with antipyrin. G. P.

**Salol.**—This is an important remedy in all kinds of specific ulcers, both soft and hard, and of glandular swellings connected with them. It causes no pain, and is as powerful as iodoform in stopping the progress of sloughs. The rate of healing is perhaps slower; but in private practice the absence of all smell, and the easiness of the application, are great recommendations. It is used, mixed with starch, in the proportion of 2 to 1. Except for rheumatism and bladder troubles, it has little value when used internally.—SCHWIMMER, *Wiener medicinische Wochenschrift*, Nos. 3–9, 1889. G. P.

**Phenyl Urethan.**—This has been recommended by Guaconnini as a new antipyretic, antirheumatic, and analgesic. It is a product of the aromatic group of carbon compounds. It forms a white crystalline powder, insoluble in water, but easily in concentrated alcohol. He considers it a powerful antipyretic, without danger, and which has no unpleasant consequences. In doses of .5 of a gramme it reduces the temperature 1° to 3° Cent., and this lasts for nine to ten hours, causing profuse sweating. The temperature falls in from 20 to 40 minutes after administration. He regards it also as an active and painless remedy for chronic articular rheumatism, reducing both the pain and swelling. Doses of .5 of a gramme are sufficient.—*Zeitschrift des Apothekerverein*, November 10th, 1889. G. P.

**The Mechanical Treatment of Locomotor Ataxia.**—Since Charcot took up the suspension treatment of tabes according to the method of Motchutkowsky, every neurologist in Christendom has experimented with it, and contributed to current literature his favourable or unfavourable views as to its value. As is often the case with remedies advised in incurable diseases, it has had transitory beneficial effects in some patients; it has been followed by no result whatever in others, and in a few it has been productive of evil, and even of fatal, consequences. A large array of devices for the purpose of stretching the spine has been placed upon the market, from a modification of the mediæval inquisitorial rack by a Vienna physician to the comparatively simple gibbet of Sayre. In many office corners the latter now stands idly by, a mute monument to many buried therapeutical hopes, or a three-legged mendicant acting as an office-servitor.

The practice of suspension for a minute or two or three, several times a week, does not seem to have accomplished everything that it promised; but as a passing fashion it has emphasised the fact that considerable good may result in many cases of disease of the spinal cord from the mere mechanical manipulation of the flexible canal containing it. It has furthermore led a number of medical troglodytes to ferret out from the dark caverns of literature old and similar methods, lost to sight until now or forgotten.

Branting, almost half a century ago, was accustomed to treat tabes by massage along the back while the patient was hanging by his hands to a horizontal bar; and in later works upon this subject, such as Hartelius's *Sjukgymnastik*, various flexing and twisting movements are recommended to be combined with massage in the treatment of this disorder. Some of the European mechanico-gymnastic institutions have long employed different species of friction and vibration for the same purpose.

Professor von Jürgensen has just called attention to a system of mechanical treatment begun by the German orthopædist, Hessing, more than fifteen years ago (*Medicinisch-chirurgisches Centralblatt*, January 3rd, 1890). Instead of stretching the spine for a few minutes at long intervals according to the Russian method of suspension, Hessing has supported and extended it day and night year after year by means of an accurately-fitted corset with flexible and extensible steel supports between the fixed points of the shoulder and pelvis on each side. It is maintained that extension of the spine may be induced in this way; but there is good ground for the statement of some orthopædists that apparatus of this kind can never be made to assume more than a quarter of the weight of the body borne by the spinal column. The shoulders are not fixed points; but, on the contrary, very movable and uncertain.

Hessing's method has been productive of improvement in cases of locomotor ataxia to about the same degree as when they have been treated by other mechanical means. There is probably some one underlying principle in all these systems; but exactly what change is brought about in the morbid process in the spinal cord by their employment, it is far from easy to determine. Release of compressed nervous elements by the stretching of sclerotic tissues, improvement of the circulation in the blood-vessels and lymph-channels, and various other hypothetical influences have been adduced to explain the somewhat obscure phenomenon.

As some cases of tabes are susceptible of amelioration by any of the methods described, and as in all of them the fundamental object seems to be to attain a certain degree of extension of the spine, perhaps it would be best to abandon the more complicated methods and apparatus, and rely upon simple exercises capable of fulfilling the same purpose, such as hanging from a horizontal bar, bending over to touch the toes with the fingers while the legs are straight, and rotatory movements of the spinal column.—*New York Medical Journal*, March 8th, 1890. L. M. G.

### HYGIENE.

**Sulphur Wcrthless for Fumigation.**—It appears that the prevailing method of disinfection by means of burning sulphur is considered by some of the leading bacteriologists as of less value than it has heretofore been considered. Dr. J. G. Johnson read a paper before the Kings County Medical Society on the 17th ult., in which he stated that he had proved the present system of fumigation as worthless for the destruction of disease-germs; that the fumes of burning sulphur do not penetrate woollens as disease-germs do. He also stated that he had propagated diphtheria from the clippings of blankets after they had undergone a thorough process of fumigation by burning sulphur. Dr. Prudden, of the New York City Board of Health, appears to have come to the same conclusion, and in both New York and Brooklyn currents of steam are to be recommended for disinfecting purposes instead of burning sulphur.—*The Medical Record*, January 4th, 1890.

